# Porphyrin-Based Covalent Organic Frameworks: Design, Synthesis, Photoelectric Conversion Mechanism, and Applications

**DOI:** 10.3390/biomimetics8020171

**Published:** 2023-04-21

**Authors:** Xiaoyu Li, Chuanyin Tang, Li Zhang, Mingyang Song, Yujie Zhang, Shengjie Wang

**Affiliations:** College of Chemistry and Chemical Engineering, China University of Petroleum, Qingdao 266580, China

**Keywords:** bio-inspired, porphyrin-based COFs, photoelectric conversion mechanism, photocatalysis, phototherapy

## Abstract

Photosynthesis occurs in high plants, and certain organisms show brilliant technology in converting solar light to chemical energy and producing carbohydrates from carbon dioxide (CO_2_). Mimicking the mechanism of natural photosynthesis is receiving wide-ranging attention for the development of novel materials capable of photo-to-electric, photo-to-chemical, and photocatalytic transformations. Porphyrin, possessing a similar highly conjugated core ring structure to chlorophyll and flexible physical and chemical properties, has become one of the most investigated photosensitizers. Chemical modification and self-assembly of molecules as well as constructing porphyrin-based metal (covalent) organic frameworks are often used to improve its solar light utilization and electron transfer rate. Especially porphyrin-based covalent organic frameworks (COFs) in which porphyrin molecules are connected by covalent bonds combine the structural advantages of organic frameworks with light-capturing properties of porphyrins and exhibit great potential in light-responsive materials. Porphyrin-based COFs are expected to have high solar light utilization, fast charge separation/transfer performance, excellent structural stability, and novel steric selectivity by special molecular design. In this paper, we reviewed the research progress of porphyrin-based COFs in the design, synthesis, properties, and applications. We focused on the intrinsic relationship between the structure and properties, especially the photoelectric conversion properties and charge transfer mechanism of porphyrin-based COFs, and tried to provide more valuable information for the design of advanced photosensitizers. The applications of porphyrin-based COFs in photocatalysis and phototherapy were emphasized based on their special structure design and light-to-electric (or light-to-heat) conversion control.

## 1. Introduction

Overreliance on fossil fuels leads to growing energy crises and environmental problems. It is imperative to develop clean and renewable energy, such as solar energy, instead of traditional fossil fuels. Organisms, including green plants and certain microorganisms, show great skill in solar energy utilization, in which solar energy is converted to chemical energy and stored in carbohydrates. Inspired by natural photosynthesis, photocatalytic conversion for solar water splitting and CO_2_ reduction to produce hydrocarbon fuels has been considered one of the most promising ways to solve the increasingly serious energy and environmental problems [1,2,3,4]. Since photocatalyst plays a pivotal role in the photocatalysis reaction, the design and preparation of highly efficient photocatalysts are important to improve solar energy utilization and photoelectric or photochemical conversion efficiency. An excellent photocatalyst requires not only light response in a wider wavelength range and high light absorption coefficient but also high photoelectric conversion and separation/transfer efficiency of charge carriers in the same substance and interface, appropriate electronic energy level, and redox gradient [5,6].

Since Fujishima and Honda first reported the photocatalytic decomposition of water on TiO_2_ electrodes in 1972 [5], many types of photocatalysts, including inorganic and organic photocatalysts, have been explored [7,8,9,10,11,12,13,14,15,16,17,18,19,20,21,22,23]. Inorganic semiconductors represented by metal oxides such as TiO_2_ have the virtues of high stability and low price. However, certain inherent defects, such as wide band gap and mismatch of conduction/valence band position, result in poor visible light utilization and low quantum yield, which limit their photocatalytic performance [16,24,25,26,27]. Different from inorganic semiconductors, organic photocatalysts have abundant structural units and flexible designability. For example, plants show excellent solar light utilization dependent on the light-capturing ability of chlorophyll. As is known, chlorophyll is a derivative of porphyrin, and its large conjugated heterocyclic structure endows it with excellent photosensitivity. At the same time, nitrogen atoms in the porphyrin ring can coordinate with different metals. Such unique structures give them high versatility and adjustability [28,29]. Inspired by natural photosynthesis, it is expected to obtain stable and efficient photocatalysts by introducing porphyrin compounds into the photocatalytic system [30,31]. 

However, the intrinsic gap between the B and Q bands of porphyrin monomers locates in the strongest output range of the sun’s total irradiance spectrum (450–550 nm), which limits their light-capturing ability. Meanwhile, low charge carrier separation and transmission efficiency limit the development of porphyrin-based photocatalytic systems [32,33]. Chemical modification or combination with other dyes can improve their absorption in the visible light region. As is known, aggregation of porphyrin molecules can change their light absorption behavior. H-aggregation (face-to-face) leads to the blue shift, while J-aggregation results in the red shift. Therefore, an alternative method for changing the spectral properties is to regulate their aggregation state. J-aggregation of porphyrin leads to the red shift of the absorption maximum to the higher energy region of sunlight. Moreover, the J-aggregation of porphyrin allows the photogenerated electrons to spread over the J-aggregates [34,35,36,37,38,39,40,41,42]. Many efforts had devoted to constructing porphyrin J-aggregates, and we know that their photocatalytic ability can be improved by introducing structural regulatory reagents [31,43,44,45,46,47], replacing or modifying peripheral groups [48,49,50,51], complexing [52,53], choosing solvents appropriately [54,55], or changing the properties of solution [56,57,58,59]. Unfortunately, the J-aggregates of porphyrin are vulnerable to environmental conditions such as pH and ionic strength because most J-aggregates are stabilized by intermolecular electrostatic interactions. Additionally, defects in the aggregates limit the delocalization of photoinduced electrons and result in lower transfer efficiency. Therefore, more efforts are needed to construct stable and efficient porphyrin-based photocatalytic systems.

Compared to the porphyrin J-aggregates stabilized by electrostatic interactions, porphyrin-based polymers connected by covalent bonds are expected to be more stable. Especially for those connected with conjugated bonds, the conjugate structure allows the photoinduced electrons to delocalize along the polymer chain, which improves the transport rate of carriers. Porphyrin-based covalent organic frameworks (COFs) possess highly conjugated structures and π-π packing and unique performances in electron transport by regulating the porphyrin units at the molecular scale [60,61]. At the same time, the material has functional designability and structural controllability, easy to adjust and optimize the band structure and surface property of porphyrin-based COFs at the molecular level, thus enabling efficient light absorption and charge separation [62,63,64,65]. Corresponding to their ordered structure, porous nature, and functional construction units, porphyrin-based COFs have widespread applications in gas storage/separation [66,67], catalysis [68,69], sensing [70,71], energy storage [72,73], and phototherapy [74,75]. 

The construction of porphyrin-based COFs using porphyrins as the light-capturing units provides a unique solution for the stable and efficient porphyrin-based photocatalytic system and exhibits great potential in photocatalytic degradation of organic pollutants, hydrogen production, inhibition of bacteria, and other aspects [62,76,77,78,79]. Through molecular design and incorporation of special functional groups, porphyrin-based COFs can be used in biomedical fields such as photodynamic therapy and photothermal therapy, indicating great values of porphyrin-based COFs in the conversion and utilization of light.

## 2. Molecular Design of Porphyrin-Based COFs

As photocatalysts, light absorption capacity, separation, and transport of photogenerated charge carriers are important to their photocatalytic properties. In porphyrin-based COFs, porphyrin groups linked in the form of covalent bonds can improve their stability, crystallinity, and catalytic activity during photocatalysis [80]. In addition, appropriate orbital energy levels and band gaps can be obtained by regulating porphyrin units, organic ligands, and conjugate bonds. Therefore, porphyrin-based COFs will be expected to have both excellent light-harvesting ability and efficient charge separation and transport behavior. Interestingly, the introduction of a donor–acceptor (D–A) pair into the conjugated structures can further enhance light capture and facilitate the separation of photogenerated charges [80]. Two key issues should be considered during the design and synthesis of porphyrin-based COFs: the first is the design of the building blocks, namely the structure of the building blocks and connection mode. The second is the synthesis method, which determines if the desired frameworks can be obtained [81,82].

### 2.1. Construction of Porphyrin-Based COFs

**Figure 1:**
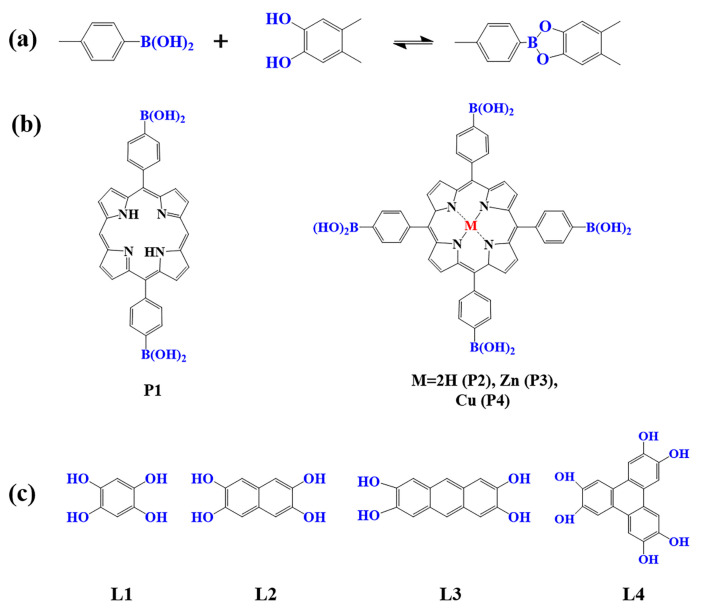
(**a**) Schematic illustration of borate-linked porphyrin-based COFs; (**b**) Frequently used monomers containing boron hydroxyl and (**c**) phenolic hydroxyl groups.

**Figure 2:**
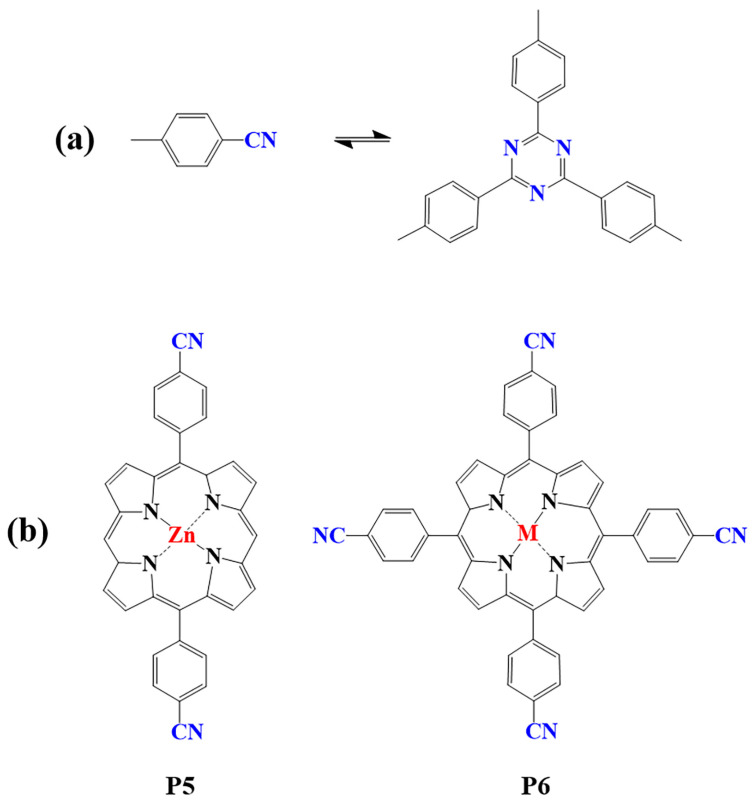
(**a**) Schematic illustration of triazine-linked porphyrin-based COFs; (**b**) Frequently used monomers.

**Figure 3:**
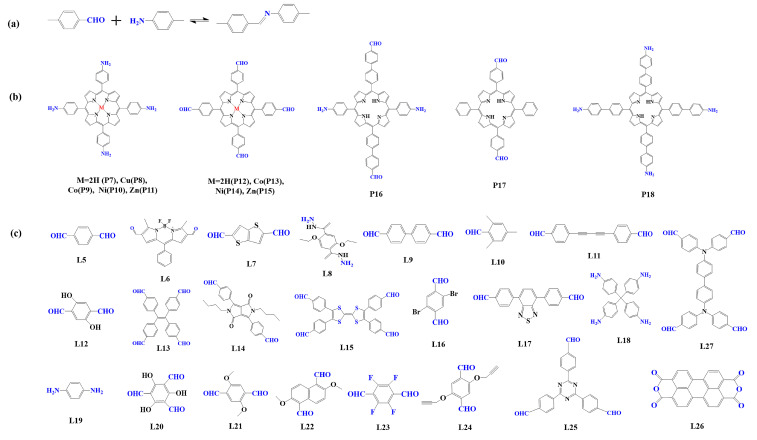
(**a**) Schematic illustration of imine-linked porphyrin-based COFs. (**b**) Frequently used monomers containing amino and (**c**) aldehyde groups.

**Figure 4:**
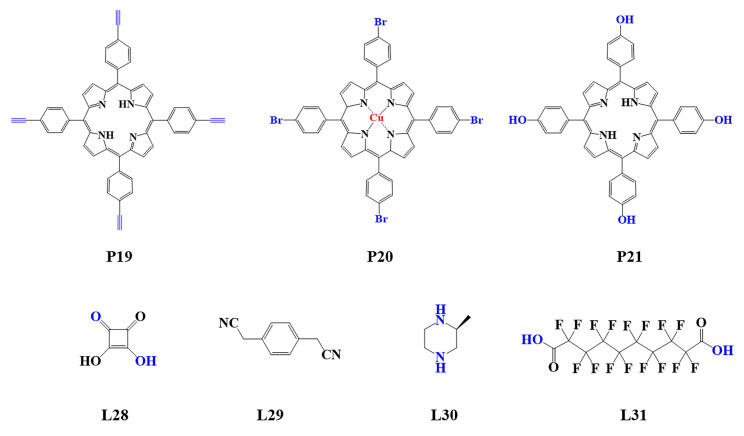
Monomers used in the synthesis of porphyrin-based COFs.

The preparation of porphyrin-based COFs is similar to those of other COFs. Borate condensation reaction (Figure 1a), triazine condensation reaction (Figure 2a), and imine condensation reaction (Figure 3a) are often used to construct porphyrin-based COFs [83]. The involved reactions are reversible and thermodynamically controlled [84,85]. There are error-checking and self-healing processes during the formation of the reversible covalent linkages [86,87,88], resulting in ordered and thermodynamically stable porphyrin-based COFs [89,90]. Additionally, other reactions, including the Yamamoto homo-coupling reaction [91], cubic acid amino condensation reaction [92], and a pot of esterification reaction [93], had been used to prepare porphyrin-based COFs (Figure 4).

#### 2.1.1. Construction of Borate-Linked Porphyrin-Based COFs

As a common reaction in organic synthesis, borate bonds generated from the condensation of borate acid and catechol derivatives are widely used to construct porphyrin-based COFs (Figure 1a). For example, porphyrin monomers containing *p*-dihydroxy boron (P1) and tetra-dihydroxy-beryl groups with different core structures (P2–P4) are condensed with 1, 2, 4, 5-tetrahydroxy benzene (L1–L4) to construct a series of borate porphyrin-based COFs [94,95,96,97]. Such porphyrin-based COFs with B–O linkages showed high crystallinity, special pore size, and large surface areas. However, compared to the porphyrin-based COFs linked by imine and triazine bonds, the COFs with borate-linkage are less stable in acidic and basic aqueous solutions, which limits their application in photocatalysis to some extent [83].

#### 2.1.2. Construction of Triazine-Linked Porphyrin-Based COFs

Porphyrin-based covalent triazine frameworks are formed by cyclotrimerization of porphyrin monomers with nitrile groups (Figure 2a). The strong aromatic group in the triazine unit and ring fusion of C=N bonds endow them with high chemical stability. However, these porphyrin-based COFs are usually amorphous and lack long-range molecular orderings due to the nonplanar trimerization route [98]. In addition, only limited members of triazine porphyrin-based COFs were reported due to the lack of suitable monomers (P5, P6) [83,99,100,101,102,103].

#### 2.1.3. Construction of Imine-Linked Porphyrin-Based COFs

Imine condensation reaction (Schiff base reaction) is the most widely used reaction in the synthesis of COFs, which is formed by the condensation of the amino group and aldehyde group (Figure 3a) [83]. The reaction is reversible, and it is easy to form a regular and ordered crystalline structure in COFs. In 2011, Yaghi et al. [95] synthesized the first imine bond-linked porphyrin COFs (COF-366) by the condensation of 5, 10, 15, 20-tetrad -(4-(amino) phenyl)-porphyrin (TAPP, P7) with p-phenyl formaldehyde (L5) under solvothermal condition, in which the carrier mobility reached 8.1 cm^2^ V^−1^ s^−1^. From then on, researchers prepared various imine bond-linked metal porphyrin-based COFs [104,105,106] by using different metal porphyrin monomers. The results showed that their photophysical and electronic properties could be regulated by the core metal ions. At the same time, to construct photocatalysts with high photocatalytic performance, researchers prefer to select monomers with electron-donating or accepting properties [62,107,108].

### 2.2. Synthesis of Porphyrin-Based COFs

The synthesis methods, structures, and properties of porphyrin-based COFs are dependent on the adopted preparation procedures. Till now, several synthetic methods, including solvothermal, microwave synthesis, and ionothermal methods, have been adopted to prepare porphyrin-based COFs. Among them, the solvothermal method is most widely used because of its high degree of universality [109]. Regular COFs were produced through the process of dissolution and recrystallization of raw materials in a closed pressure vessel at a certain temperature [86]. Great efforts have been made to optimize the synthesis conditions for the preparation of high-quality COFs. The crystallinity of porphyrin-based COFs is very sensitive to the reaction conditions. Therefore, their crystal state, morphology, pore size distribution, and other properties would be related to solvent, catalyst, temperature, reaction time, pressure, and other reaction parameters [110,111,112]. At the same time, the intrinsic relationship between the structure and property also attracted considerable attention and was usually used to direct the regulation of their photocatalytic properties [83,90].

Microwave synthesis showed several advantages over solvothermal methods, such as high reaction speed, high heading efficiency, and well-distributed heating [113], resulting in the formation of cleaner products with higher yields within less time. Compared to traditional thermal-assisted methods, microwave-assisted methods have the advantages of easy control, fast kinetic energy speed, high heating efficiency, and fast and uniform heating rate [114], which is conducive to obtaining COFs with high purity. The ionothermal method refers to the cyclization trimerization reaction in ZnCl_2_ at 400 °C using a nitrogen heterocyclic ring as the basic element [100]. The reaction is only partially reversible, resulting in less crystallinity. Moreover, harsh reaction conditions are usually required, which narrows the scope of porphyrin-based COFs.

## 3. Photoelectric Conversion Mechanism

Photoelectric conversion is crucial to a photocatalytic reaction, which generally includes two steps. First, photons are captured by the semiconductors or organic chromophores and converted to electrons, in which the spectral response range and photoelectric conversion ability of the light-harvesting unit determine the upper limit of photocatalytic activity. The band structure of semiconductor materials is generally composed of a low-energy valence band (VB) filled with electrons and an empty high-energy conduction band (CB) without occupied electrons. When the semiconductor photocatalyst is exposed to light irradiation, electrons in the VB are excited by the photons and transferred to the CB. Photogenerated electrons (e^−^) in the conduction band and corresponding holes (h^+^) in the valence band are generated. There is a band gap between the conduction band and the valence band, which is also called the forbidden bandwidth. To complete the photoelectric conversion, the energy of the photons should be greater than the band gap energy of the semiconductor photocatalysts.

**Figure 5:**
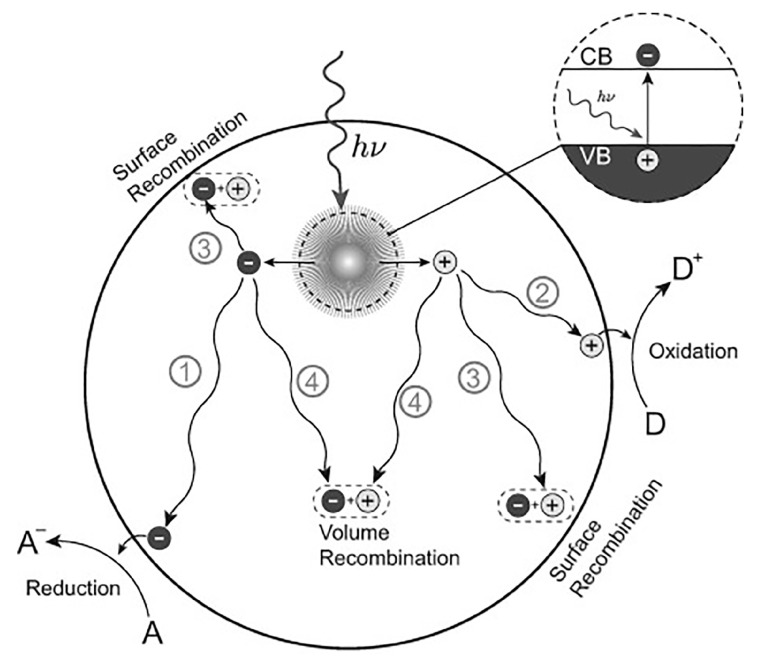
Photoinduced formation of an electron–hole pair in a semiconductor with possible decay paths. A = electron acceptor, D = electron donor [115].

The second is the separation and transmission of photogenerated carriers. Some photogenerated carriers are separated by spontaneous diffusion or the action of the electric field and move to the surface of the catalyst for a subsequent redox reaction (Figure 5, pathways ① and ②) [2]. Meanwhile, the carriers can recombine with their counterparts of opposite charges trapped on the surface (Figure 5, pathway ③), or the recombination of carriers occur in the bulk of the semiconductor (Figure 5, pathway ④) [115]. To transfer as many photogenerated carriers as possible to the surface of the catalyst, a long carrier lifetime, short diffusion distance, weak diffusion resistance, and low recombination probability are usually required [116]. This puts forward higher requirements for the design and preparation of porphyrin-based COFs.

### 3.1. Absorption of Light

**Figure 6:**
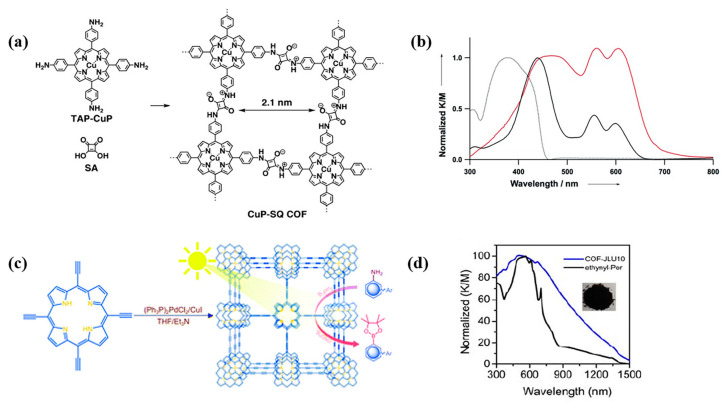
(**a**) Synthesis of CuP-SQ COF [92]; (**b**) UV-vis absorption spectra of CuP-SQ COF (red), TAP-CuP (black), and SQ (dashed line curve) [92]; (**c**) Schematic showing the synthesis of Cof-JLU10 [120]; (**d**) UV-vis spectra of CoF-JLU10 and the corresponding monomer [120].

The light-capturing ability of semiconductors depends on their band gap. According to the band theory, the band gap of COFs hinges on the energy difference between their highest occupied molecular orbital (HOMO) and lowest unoccupied molecular orbital (LUMO) energy levels, corresponding to the valence band (VB) and conduction band (CB) in inorganic semiconductors, respectively [117]. Electrons transfer from HOMO to LUMO when the photocatalyst is exposed to light irradiation. Two kinds of electron transition may occur. One is σ→σ* transition, in which the absorption is in the ultraviolet region (10–400 nm). Another transition of π→π* occurs in the visible light region (380–780 nm) [118]. Covalent linkages, especially conjugated linkages in COFs, can narrow their band gap and improve their light-harvesting capacity [119]. Moreover, the incorporation of highly conjugated structures into COFs, such as porphyrins, sulfones, and imidazole, further expanded the absorption range from ultraviolet to near-infrared region and improved their light absorption capacity [76]. For example, the porphyrin-based COFs (CuP-SQ COF), which are covalently linked by cubic acid (SQ, L28) and copper (II) 5, 10, 15, 20-tetrad (4-aminophenyl) porphyrin (TAP-CuP, P8), showed a narrow band gap (1.7 eV) and greatly enhanced optical absorption in both visible and near-infrared regions (Figure 6a), far beyond the absorption regions of the two monomers (~370 nm and ~440 nm for SQ and TAP-CuP, respectively). The widened and redshifted Soret band of CuP-SQ COF can be ascribed to the delocalization of π electron by the intramolecular conjugation effect (Figure 5b) [92]. Similarly, compared to the corresponding monomer, COF-JLU10 synthesized from 5,10,15, 20-tetra(ethynyl)porphyrins (P19) exhibited an ultra-wide absorption band from 300 nm to 1500 nm (Figure 6c,d) [120] and a band gap of 1.37 eV. These results demonstrated that the optical response range and absorption intensity of the photocatalysts could be further optimized by the formation of COFs with conjugated structures.

Compared with porphyrin monomers, the porphyrin-based COFs have greatly expanded π-conjugated structures, which significantly enhance the delocalization of π electrons, optimize their light absorption behavior, and further improve their photocatalytic activity. Additionally, different porphyrin units and functional groups are connected by different chemical bonds, which endow porphyrin-based COFs with flexible band structures (such as HOMO and LUMO position and band gap) and variable light absorption capacity [118]. Therefore, porphyrin-based COFs with desired structures and functions can be designed and regulated at the molecular level.

### 3.2. Electron Separation and Transport

#### 3.2.1. Incorporation of D–A Conjugate Structure

The incorporation of donor–acceptor (D–A) pairs into the porphyrin-based COFs can promote charge transfer along the polymer frameworks and have unique advantages in improving their photocatalytic performance. Porphyrin-based COFs (Tph-BDP), constructed from Tph (P7) and BDP (L6), are a typical D–A conjugated structure [62]. The electron transfer from Tph to BDP contributes to the effective separation of photogenerated carriers and excellent photocatalytic activity. Compared with monomeric Tph and BDP, TPH-BDP has a narrower band gap. This means that more electrons will be photoexcited and transferred into the conduction band, and the recombination of photogenerated carriers will be suppressed.

The electron conduction and carrier mobility value of TT-Por(Co)-COF (P9 + L7) with D–A structure is much higher than that of COF 366-Co (P9 + L5) without D–A structure due to their different electron transfer behavior. The charge transfer from TT (electron donor) to porphyrin cobalt (electron acceptor) in TT-Por(Co)-COF promotes the rapid transport of the photogenerated charges from porphyrin-based COFs [105]. This means that we can design and construct porphyrin-based COFs with excellent charge separation performance by incorporating D–A units.

#### 3.2.2. Utilization of Metal Coordination Center

**Figure 7:**
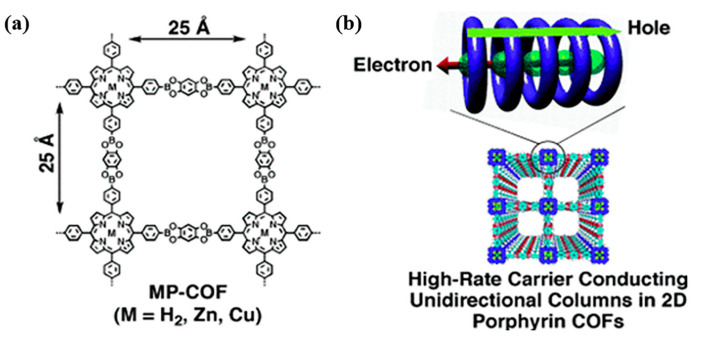
(**a**) Schematic representation of MP-COFs (M = H_2_, Zn, and Cu) [94]; (**b**) graphical representation of metal-on-metal and macrocycle-on-macrocycle channels for respective electron and hole transport in stacked porphyrin columns of MP-COFs [94].

The transport performance of charge carriers in porphyrin-based COFs can also be regulated by the metal coordination centers. A series of porphyrin-based COFs (MP-COF, M = H_2_, Zn, and Cu) had been synthesized from M-5, 10, 15, 20-tetra[4-(dihydroxyboryl)phenyl]porphyrin (M = H_2_, Zn, Cu) (P1–P4) and 5-tetrahydroxy benzene (L1). The MP-COFs with metal atom center showed high carrier transport capacity (Figure 7) owing to their two carrier transport channels in the porphyrin column; that is, macrocycle-on-macrocycle and metal-on-metal channels correspond to the holes and electrons transport, respectively, through which the holes and electrons are transported within the COFs [94]. These results indicate that the metals in the porphyrin ring play an important role in the formation of electron conduction channels and the regulation of the ease of carrier motion. This can be used to design and construct porphyrin-based COFs with excellent transport performance of photogenerated charges and high catalytic activity.

Similarly, MPor-DETH-COF (M = H_2_, Co, Ni, Zn) (L8 + P12–P15) is a series of 2D porphyrin-based COFs with the same configuration. They showed different capabilities in carrier separation and hydrogen production capabilities relying on M in the porphyrin ring. ZnPor-DETH-COF exhibited the highest hydrogen production rate of 413 μmol g^−1^ h^−1^ owing to their tailored charge-carrier dynamics via molecular engineering. The macrocycle-on-macrocycle and metal-on-metal channels in porphyrin columns were expected to play an important role in carrier separation and transport. Upon light irradiation of the porphyrin π ring, the photogenerated electrons migrate along metal-metal channels, while the photogenerated holes transfer mainly through the macrocyclic-macrocyclic channels. As there are no metal atoms in the inner ring of H_2_Por-DETH-COF, both electrons and holes transport through the macrocycle-on-macrocycle channel, which increases the recombination of photogenerated electron-hole pairs. CoPor-DETH-COF and NiPor-DETH-COF presented higher photocatalytic activity due to the presence of metals in the ring, in which ligand-to-metal charge transfer (LMCT) inhibited the transfer of holes in the macrocycle-on-macrocycle channel and reduced the recombination of photogenerated carriers [106]. Therefore, the photophysical property and charge transfer behavior of porphyrin-based COFs can be regulated at the molecular level by the incorporation of different metals in porphyrin. The results provided an easy but effective strategy for advanced porphyrin-based COFs photocatalysts by precisely controlling the charge transfer channels.

#### 3.2.3. Shorten Carrier Transfer Path

**Figure 8:**
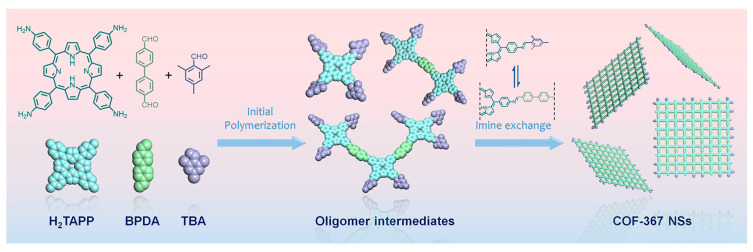
Schematic illustration of the synthesis procedure for the COF-367 NSs [122].

Bulk or powdered COFs are often obtained by traditional synthesis methods (such as the solvothermal method). By contrast, covalent organic nanosheets (CONs) are a class of two-dimensional COFs with fewer layers that shorten the carrier transport paths and exhibit unique optical and electronic properties [121]. Based on the porphyrin-based COFs with imines linkages such as COF-366 (P7 + L1) and COF-367 (P7 + L9), Jiang et al. [122] developed a bottom-up method for ultrathin COF nanosheets (Figure 8). The introduction of 2, 4, 6-trimethylbenzoic aldehyde (TBA, L10) into the reaction system prevented the axial π-π stacking of the COF NS from forming bulk COFs and, at the same time, allowed the anisotropic growth of the COF NSs along the planar direction, thus ensuring the formation of non-stacked ultrathin 2D COF NSs. Using this technique, ultrathin porphyrin-based nanosheet COF-367-Co NSs (P9 + L9) with a thickness of 1.1 ± 0.1 nm were prepared and showed a much higher photocatalytic rate than the bulk COF-367-Co.

In theory, the overlap of the π-π electron clouds between the adjacent layers and the conjugated bonds within the layers enable rapid carrier transport along the skeleton of COFs. Therefore, the charge carrier conduction in COFs mainly depends on the transport pathway provided by π-π interactions [123,124,125]. However, the degree of intralaminar conjugation is limited by the steric hindrance [126,127,128,129], and the interlaminar stacking is often not tight enough. These make the actual conductivity of COFs relatively insufficient and limit their further application in the field of photoelectric conversion. Ultrathin COF nanosheets would shorten the transport path of charge carriers and improve their photocatalytic activity.

Moreover, the building of conjugated bonds between COF layers may provide an opportunity to further improve their photo and electrical properties. Electron transmission between layers can also be constructed through interlayer conjugated polymerization to strengthen the overall conjugation degree of the skeleton network [125,130,131,132]. For example, conjugated COF (TAPP-COF-P, P7 + L11) [125] connected by an enyne chain exhibited a broad absorption at 700 to 1200 nm, which exhibited obvious improvement in near-infrared photothermal conversion and photoacoustic imaging ability compared to its corresponding imine-linked COF. Such interlayer connection mode by the olefin chains provides a new opportunity for the development of photoelectric devices and porphyrin-based COF photocatalysts.

## 4. Photocatalytic Application

### 4.1. Photocatalytic Hydrogen Production

Hydrogen energy is regarded to be an efficient, environmentally friendly, and clean energy source owing to its high energy density and pollution-free combustion products. At present, hydrogen is mainly obtained from coal and natural gas through a conversion reaction, at the cost of large quantities of energy consumption and heavy emission of carbon dioxide. Photocatalytic hydrogen evolution is an environmentally friendly and sustainable technology in which only sunlight and water are consumed, and no carbon dioxide is produced. This technology has received wide attention because it meets the increasing demands for clean and sustainable energy [133,134]. Porphyrin-based COFs exhibit excellent visible light responsiveness, controllable electronic band structures, and efficient separation/transfer of charges as well as great potential in photocatalytic hydrogen evolution.

Corresponding to their excellent charge separation capability, photocatalytic hydrogen evolution was completed in the presence of porphyrin-based COFs (MPor-DETH-COF, M = H_2_, Co, Ni, Zn) under visible light irradiation, in which Pt nanoparticles were in situ generated and used as a co-catalyst, and triethanolamine was used as the electron donator [106]. The average rates of hydrogen evolution over H_2_Por-DETH-COF, CoPor-DETH-COF, NiPor-DETH-COF and ZnPor-DETH-COF within 10 h were determined to be 80 μmol g^−1^ h^−1^, 25 μmol g^−1^ h^−1^, 211 μmol g^−1^ h^−1^, and 413 μmol g^−1^ h^−1^, respectively. The introduction of different coordination metals endows them with various charge transfer abilities and photocatalytic hydrogen evolution performance.

One of the porphyrin-based COFs (DhaTph, P7 + L12) was exfoliated into 1 nm thick dispersed nanosheets (e-CON) to shorten the carrier transport path. DhaTph showed improved photocatalytic ability in hydrogen evolution. The apparent quantum yields are 5.1% at 420 nm and 2.3% at 780 nm, respectively [135]. In addition, the incorporation of metal porphyrin into the COFs (DhaMTph, M = Cu, Ni; P8, P10 + L12) also improved their photocatalytic activity [136]. Similarly, the photocatalytic activity of the dispersed nanosheets of DhaMTph can be further improved by shortening the transfer path of charges. Compared to the bulk porphyrin-based COFs, the dispersed nanosheets not only enhance their charge transfer capacity but also increase the specific surface area of the photocatalysts and reactive sites. The introduction of coordination metals has further improved their photocatalytic activity. This provides a new idea for improving the photocatalytic performance of porphyrin-based COFs. However, porphyrin-based COFs have some shortcomings, such as lower yield of hydrogen evolution and complex preparation processes compared to other photocatalytic catalysts. Hence, it needs more efforts to improve the efficiency of light utilization and carrier separation and further reduce its production cost.

### 4.2. Photocatalytic Carbon Dioxide Reduction

Photocatalytic CO_2_ reduction into hydrocarbon fuels or chemicals is an effective strategy to obtain clean fuels and, at the same time, alleviate the greenhouse effect and promote the carbon cycle [137,138]. However, most of the carbon dioxide photoreduction systems require additional photosensitizers, sacrificial agents, electron mediators, and noble metal co-catalysts, which wastes the oxidizing power of photoinduced holes and increases the cost of the conversion and the difficulty in the post-treatment [139]. Therefore, how to obtain efficient and highly selective photocatalysts is still a challenge for CO_2_ photocatalytic reduction. Porphyrin-based COFs have attracted wide attention in visible-light-driven CO_2_ reduction due to their rich functionality, designability, high durability, and recyclability [100,104,140,141].

Porphyrin-base COFs with D-A pairs (MP-TPE-COF, M = H_2_, Co, Ni; P7, P9, P10 + L13) were used as a photocatalyst for the visible-light-driven CO_2_-CO conversion. Corresponding to their novel properties in the separation of charge carriers and electron transfer, the D-A structure in MP-TPE-COF endows them with high photocatalytic efficiency and excellent cycling stability at saturated CO_2_ or even low CO_2_ concentrations [104]. Under the same experimental conditions, using H_2_P-TPE-COF as a photocatalyst, negligible CO was generated. Compared with H_2_P-TPE-COF, CoP-TPE-COF, and NiP-TPE-COF showed higher conversion and reaction rates of CO_2_ (1575 μmol g^−1^ and 4828 μmol g^−1^, respectively). Moreover, the incorporation of metalloporphyrin also improved the selectivity of CO compared to the H_2_P-TPE-COF. In particular, the selectivity of NiP-TPE-COF for CO was as high as 93%. The results demonstrate that the D-A conjugated structure in porphyrin-based COFs and the coordination metals in the porphyrin ring have crucial roles in boosting photocatalytic activity and product selectivity. Unfortunately, additional photosensitizers and sacrificial electron donators are necessary for such photocatalytic reactions.

**Figure 9:**
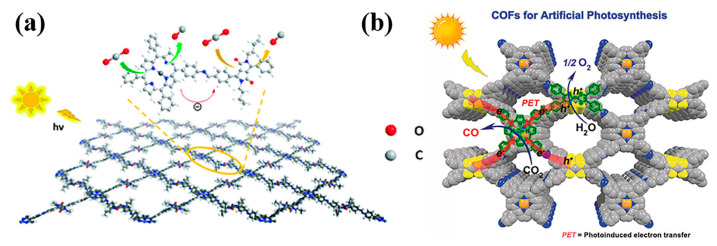
(**a**) Schematic diagram of D-A PD-COF photocatalytic reduction of CO_2_ [139]; (**b**) Schematic diagram of TTCOF-M photocatalytic reduction of CO_2_ [65].

In the absence of a photosensitizer and noble metal co-catalyst, the photocatalytic reduction of CO_2_ was completed by using the porphyrin-based COFs with D-A structures (PD-COF-23 and PD-COF-23-Ni) as the photocatalyst and triethanolamine as the sacrificial electron donator (Figure 9a) [140]. Tetra(aminophenyl)ene porphyrin (MTAPP, M = 2H, Ni) (P7, P10) was used as the electron donor, and 2,5-dibutyl-3,6-bis(4-formylphenyl)-pyrrolo[3,4-c]pyrrole-1,4-dione (DPP-CHO, L14) was used as the electron acceptor. The D-A structures contribute to charge separation and transfer. At the same time, the incorporation of metals in porphyrin rings has an important effect on the electron mobility and energy band structure regulation of porphyrin-based COFs. They both improve the photocatalytic ability of the porphyrin-based COFs to fulfill the reduction of CO_2_ without a photosensitizer and noble metal.

The structure design of electron donors and acceptors in porphyrin-based COFs has a crucial role in the photocatalytic effect. For example, electron-rich tetrathiafulvalene (TTF, L15) exhibited a superior electron donor property and rapid electron transfer behavior [140]. Using TTF as the electron donor part, the constructed porphyrin-based COFs (TTCOF-M, M = H_2_, Zn, Ni, Cu) [65] can drive CO_2_ reduction and H_2_O oxidation (the CO/O_2_ ratio is about 2:1) under visible light irradiation without additional photosensitizer, metal co-catalyst, or sacrificial agent (Figure 9b). When the COFs were exposed to visible light irradiation, the excited electrons were transferred from the TFF units to the porphyrin units via covalent bonds to participate in the reduction of carbon dioxide, while the holes left in TTF are capable of oxidizing H_2_O to O_2_. Moreover, the CO yield over TTCOF-Zn photocatalyst reached 12.33 μmol with circa 100% selectivity. This is the first report of porphyrin-based COFs for the selective photoreduction of CO_2_ using H_2_O as the electron donor. Compared with other photocatalytic system using sacrificial electron donators, this photocatalytic system can complete the photocatalytic reduction of CO_2_ with a relatively lower yield, so the photocatalytic performance of the porphyrin-based COFs needs to be further improved by rational design and other methods.

The selection of electron donor and acceptor moieties of porphyrin-based COFs has a great influence on their photocatalytic performance. The introduction of halogen groups such as bromine can change the electronic properties of organic ligands. A novel porphyrin-based COFs (TAPBB-COF) was constructed by the reaction of 2,5-dibromo-1,4-benzenedialdehyde (L16, an electron-absorbing ligand) and TAPP (P7). TAPBB-COF also showed excellent capacity in photocatalytic CO_2_ reduction using H_2_O as an electron donor [142]. Compared to COF-366 (P7 + L5) without the bromine group, the conversion rate of CO over TAPBB-COF photocatalyst was 24.6 μmol g^−1^ h^−1^, three times that catalyzed by COF-366. The valence band of TAPBB-COF increased from +0.86 V of COF-366 to +1.10 V owing to the introduction of electron acceptor units. The band gap of TAPBB-COF decreased and had a favorable redox potential for the photocatalytic reaction because there was little change in the position of the conduction band. Different from TTCOFs (L15 + P7, P8, P10, P11) in which porphyrin units functioned as electron acceptor units, porphyrin in TAPBB-COF existed as the electron donator due to the electron-withdrawing and conjugation effect of bromine. The change in the donor-acceptor structure resulted in TAPBB-COF having a higher photocatalytic capacity for CO_2_ reduction. This provides a new idea for the design and construction of efficient porphyrin-based COF photocatalysts.

### 4.3. Photocatalytic Synthesis of Organic Compounds

The application of porphyrin-based COFs in photocatalytic organic synthesis is still in its infancy compared to photocatalytic hydrogen evolution and carbon dioxide reduction. Until now, only a few relatively simple photocatalytic reactions have been reported [143]; most of them are direct oxidation reactions. Therefore, broadening the range of photocatalytic synthesis of organic compounds will achieve more meaningful goals.

Sulfoxides are indispensable intermediates in pharmaceutical, agrochemical, and other fine chemical industries [144,145]. They are produced from the selective oxidation of sulfide, which usually requires harsh oxidants and generates toxic byproducts and heavy metal wastes [146]. A_2_B_2_-Por-COF, a porphyrin-based COFs synthesized by self-condensation of A_2_B_2_-Por monomer (P16), can be used to oxidize thioanisole to sulfoxide at room temperature under visible-light irradiation using oxygen as a green oxidant [147]. The conversion efficiency was up to 96%, and selectivity was higher than 99%. The results showed that the A_2_B_2_-Por-COF photocatalyst produced electrons and holes when exposed to visible light and then produced reactive oxygen species ^1^O_2_ through the energy transfer process. Thus, sulfoxides were generated by the reaction of diphenyl sulfide and the reactive oxygen species. In addition, A_2_B_2_-Por-COF exhibited excellent catalytic performance on the Knoevenagel condensation reaction under mild conditions. This provides a new environmentally friendly and efficient way for the synthesis of sulfoxides and other organic chemicals.

**Figure 10:**
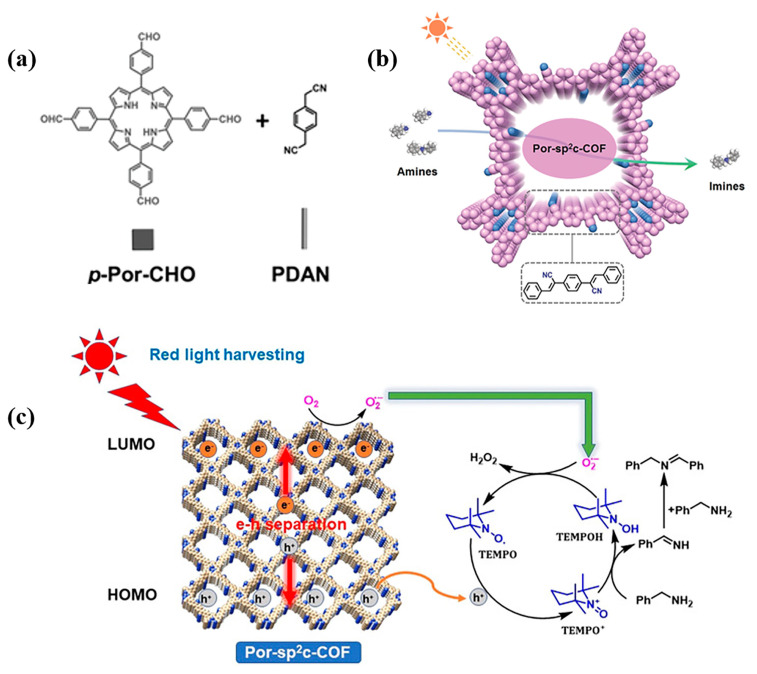
(**a**) Synthesis of Por-sp^2^c-COF [148]; (**b**) schematic diagram of Por-sp^2^c-COF photocatalytic oxidation [148]; (**c**) A plausible mechanism of visible-light-induced selective aerobic oxidation of benzylamine by cooperative Por-sp^2^c-COF photocatalysis and TEMPO catalysis [149].

Imines are a kind of important active intermediate and attract great attention in the field of medicine and biology due to their good antibacterial effect and pharmacological and biological activity. The metal-free heterogeneous photocatalyst Por-sp^2^c-COF (P12 + L29) can catalyze the oxidation of amines to imines within short times (Figure 10a,b). Taking the oxidation of dibenzylamine to N-benzylidene-benzylamine as an example, the conversion yield of this reaction was as high as 99% within 30 min, much better than that of many reported heterogeneous photocatalysts and even comparable to some metal-based heterogeneous photocatalyst [148]. At the same time, compared with the Por-COF (P12 + L19), which is connected with C=N, the C=C bond in Por-sp^2^c-COF would not be destroyed by benzylamine, which guarantees its stability under the reaction conditions. In addition, Por-sp^2^c-COF can be used to catalyze the selective oxidation of a wide range of amines under red LED illumination. This is the first report of red-light-driven conversion of amines by porphyrin-based COFs photocatalysis (Figure 10c) [149]. Both primary and secondary amines can be oxidized to the corresponding imines within 30 min with extremely high conversion rates and unprecedented selectivity.

## 5. Photocatalytic Application

**Figure 11:**
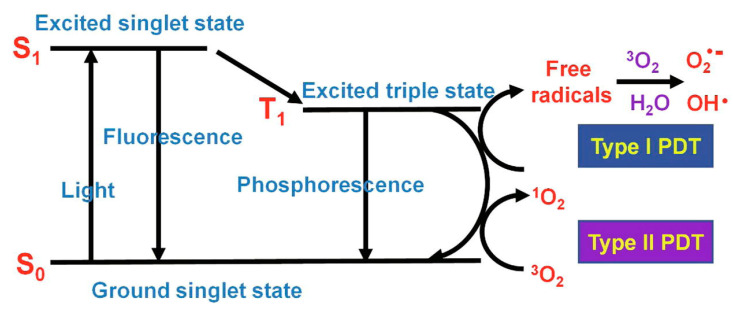
Simplified Jablonski diagram [149].

Cancer is one of the world’s public health problems. Phototherapy, including photothermal therapy (PTT) and photodynamic therapy (PDT), has become one of the most promising clinical treatments. Photosensitizers absorb photons to produce charge carriers under light irradiation, in which excited singlet electrons can undergo intersystem span to produce long-lived excited triplets and relaxation, whose energy are converted into fluorescence, heat, and/or other forms of photophysical energy (Figure 11) [150]. Owing to intermolecular collisions or aggregation, the excited electrons cannot return to the ground state, and part of the energy will be released in the form of heat. Photothermal therapy is a method of tumor therapy utilizing conversion heat. Unlike PTT, PDT involves the transfer of electron energy to oxygen and the production of reactive oxygen species (ROS) to kill tumor cells.

### 5.1. Photothermal Therapy

Due to the better tissue penetration of near-infrared (NIR) light, ideal phototherapy should have high absorption in the NIR region. Porphyrin-based COFs, which have large conjugated structures similar to organic photothermal reagents, can absorb near-infrared light and generate electrons, resulting in thermal effects occurring through nonradiative transitions [151]. For example, CCOF-CuTPP, a porphyrin-based COF prepared from copper(II) 5,10,15,20-tetrakis(4-bromophenyl)porphyrin (P20) and (S)-2-methylpiperazine (L30) [152], exhibited a considerable photothermal conversion ability under visible light irradiation and was expected to have in vitro and in vivo PTT potential [153].

**Figure 12:**
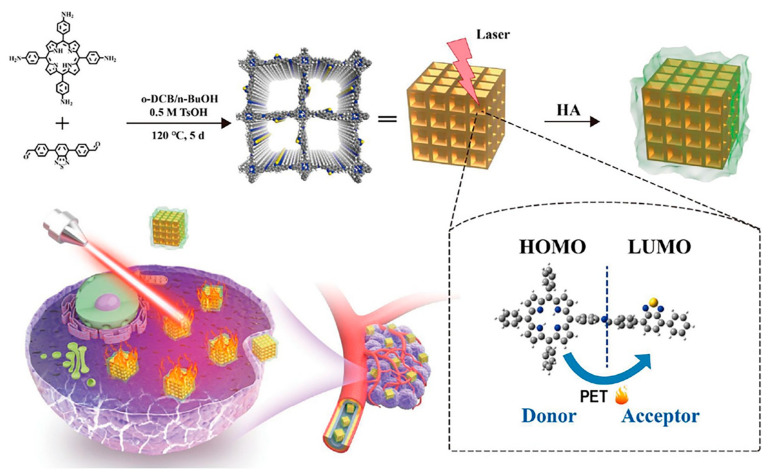
Schematic for the design and preparation of TB-COF-HA for PTT [153].

The photothermal conversion efficiency of TB-COF (P7 + L17) reached 43.65%, which is similar to that of commonly used PTT reagents [153]. TB-COFs with D-A structures undergo photoinduced electron transfer (PET) under laser irradiation, through which the absorbed light energy is converted into heat energy and cancer cells are killed (Figure 12). At the same time, hyaluronic acid (HA) coating on TB-COF can improve its biocompatibility and achieve targeted tumor therapy. In vitro and in vivo experiments have confirmed the satisfactory photothermal efficacy of TB-COF-HA.

Photothermal therapy kills tumor cells by hyperthermia (>50 °C) in the presence of photothermic agents, which inevitably damages surrounding healthy tissues. The composite of porphyrin-based COFs (P7 + L12) and gambogic acid (GA) can improve the photothermal treatment efficiency at low temperatures (<45 °C) [154], resulting in effective cancer treatment through mild photothermal therapy. Porphyrin-based COFs have good biocompatibility and photothermal conversion efficiency. However, there are few reports about the porphyrin-based COFs used for photothermal therapy. It needs more effort to explore and develop more efficient porphyrin-based COFs for photothermal therapy.

### 5.2. Photodynamic Therapy

Porphyrins and their derivatives can produce singlet oxygen (^1^O_2_) under light irradiation and have been widely studied as photosensitizers for photodynamic therapy [155]. Traditional photosensitizers usually have problems such as poor tumor selectivity, limited light penetration, and poor ROS generation effects. The highly conjugated structure of porphyrin-based COFs and ordered π-π stacking between layers endows them with good light absorption and singlet oxygen generation ability, which effectively overcomes the problems of traditional photosensitized agents. At the same time, it can effectively avoid the self-aggregation of porphyrin molecules in an aqueous medium which usually reduces its reaction activity. The easy modification in structure and the large specific surface area also effectively increase the active site, which can further improve its reaction activity. Therefore, porphyrin-based COFs have great potential in photodynamic therapy.

Lang et al. [156] reported a preliminary study of the preparation of a 3D-TPP COF (P17 + L18) by Schiff base reaction and its photodynamic antibacterial effect. The 3D-TPP COF not only has a high local loading concentration of porphyrin but also has a high production rate of singlet oxygen similar to that of porphyrin molecules in a good solvent.

However, the molecular sizes of porphyrin-based COFs are usually too large to be used in organisms. To solve this problem, Qu et al. [157] constructed porphyrin-based COF nanodots (TAPPT-DHTA-COF, P7 + L12) and used them as photosensitizers for PDT. The porphyrin-based COF nanorods showed high reactive oxygen generation ability and thus improved PDT efficiency in vitro and in vivo. Because the COF nanodots are ultra-small in size (3–4 nm), they can be removed from the body by renal filtration without causing long-term toxicity. Moreover, PEG coating further enhanced the biocompatibility and biostability of COF nanodots. The PEG-coated porphyrin-based COF nanodots have good photodynamic efficiency in cancer cell therapy and in vivo tumor suppression.

In addition, visible light can be attenuated by endogenous biomolecules such as hemoglobin and melanin, resulting in limited tissue penetration [79,156,158,159,160]. The high tissue penetration of NIR light allows us to achieve in situ PDT with enhanced therapeutic effect by the combination of porphyrin-based COFs with excellent NIR exciting materials. For example, UCNP@COF composites consist of porphyrin-based COFs (P7 + L12) and lanthanide-doped upconversion nanoparticles (UCNP) [161] after UCNP emits red light of 654 nm under the irradiation of 980 nm near-infrared light, which facilitates the porphyrin photosensitizer to produce ^1^O_2_.

**Figure 13:**
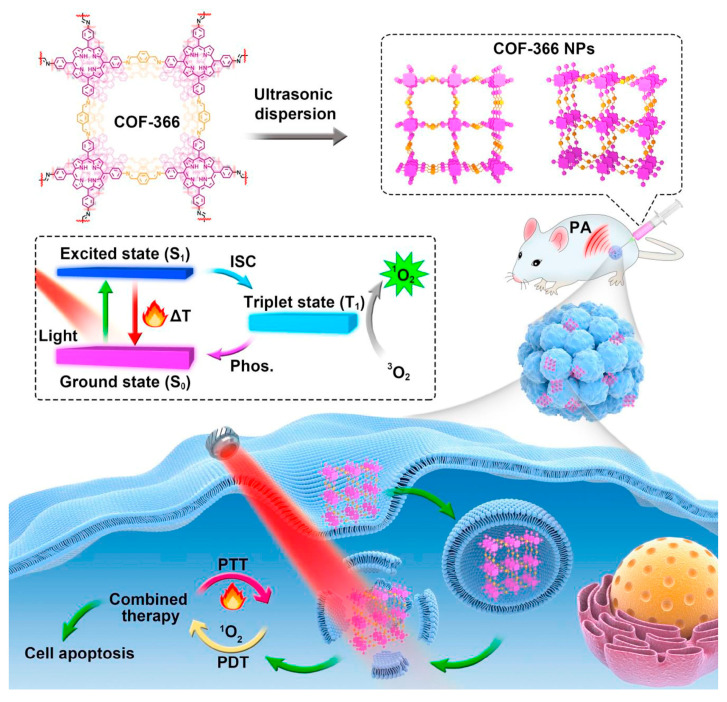
Schematic diagram of combined PDT/PTT therapy for COF-366 NPs [162].

Furthermore, the combination of PDT and PTT of porphyrin-based COFs can be used to enhance the anti-tumor effect. Porphyrin-based COF nanoparticles, COF-366 NPs [162] (P7 + L5), are capable of generating ROS and heat under single-wavelength light irradiation. Therefore, it can be used as a photoactive reagent in combining PDT with PTT in vivo (Figure 13). The anti-degradation ability of porphyrin-based COFs enhances their biosafety, and PA imaging can be used for precise anti-tumor therapy in vivo. COF-366 NPs have strong absorption in the near-infrared light region, which may result in photothermal transformation. At the same time, the ordered conjugated structure of porphyrin-based COFs extensively increases the light absorption capacity, reduces the degree of photogenerated carrier recombination, and enhances the efficiency of phototherapy.

With the development of new COFs, the applications of COFs in photothermal therapy, photodynamic therapy, and drug delivery are increasing gradually. Porphyrin-based COFs have exhibited great potential in the biomedical field due to their unique photoelectric and photothermal properties and provide a powerful means to solve the challenges in biomedical fields. However, most of COFs faced many challenges, including complex preparation procedures and long-term biocompatibility, which limit their practical application to some extent. Therefore, considerable efforts should be devoted to designing and constructing porphyrin-based COFs with definite morphology and desirable properties.

## 6. Summary and Perspective

In summary, porphyrins have excellent light absorption ability and long excited-state lifetimes, while COFs have a regulable structure and high chemical stability. Porphyrin-based COFs using porphyrins as the functional unit are expected to possess unique photoelectric conversion and charge separation/transfer behavior. On the one hand, the electron transport orbit is formed by conjugated chemical bonds and π-π interaction stacking, which makes it have a long excited-state lifetime and higher carrier mobility. On the other hand, the structure-activity relationship of the COFs can be explored in detail by using the reliability of COFs and their accurately analyzable structures. This is helpful in the design of efficient and stable photocatalysts similar to the natural photosynthesis system. In addition, COFs are usually composed of only light atoms (such as C, H, O, N, and B, etc.), which predict better biocompatibility and great potential for biomedical applications.

Although porphyrin-based COFs have shown many advantages and made great progress in recent research, their preparation procedures are complicated, and their performances need to be further improved. Design at the molecular level and even at the electron level will improve the electron-hole separation and migration rate, reduce the recombination probability of the photogenerated carriers, and thus improve the conversion and utilization of light energy. In addition, more attention should be paid to the improvement of the NIR light absorption, photothermal transformation efficiency, and high singlet oxygen yield, thus achieving an effective phototherapy effect.

## Data Availability

Data sharing is not applicable to this article as no new data were created or analyzed in this study.
